# Electrospray sample injection for single-particle imaging with x-ray lasers

**DOI:** 10.1126/sciadv.aav8801

**Published:** 2019-05-03

**Authors:** Johan Bielecki, Max F. Hantke, Benedikt J. Daurer, Hemanth K. N. Reddy, Dirk Hasse, Daniel S. D. Larsson, Laura H. Gunn, Martin Svenda, Anna Munke, Jonas A. Sellberg, Leonie Flueckiger, Alberto Pietrini, Carl Nettelblad, Ida Lundholm, Gunilla Carlsson, Kenta Okamoto, Nicusor Timneanu, Daniel Westphal, Olena Kulyk, Akifumi Higashiura, Gijs van der Schot, Ne-Te Duane Loh, Taylor E. Wysong, Christoph Bostedt, Tais Gorkhover, Bianca Iwan, M. Marvin Seibert, Timur Osipov, Peter Walter, Philip Hart, Maximilian Bucher, Anatoli Ulmer, Dipanwita Ray, Gabriella Carini, Ken R. Ferguson, Inger Andersson, Jakob Andreasson, Janos Hajdu, Filipe R. N. C. Maia

**Affiliations:** 1 Laboratory of Molecular Biophysics, Department of Cell and Molecular Biology, Uppsala University, Husargatan 3 (Box 596), SE-75124 Uppsala, Sweden.; 2 European XFEL GmbH, Holzkoppel 4, 22869 Schenefeld, Germany.; 3 Chemistry Research Laboratory, Department of Chemistry, Oxford University, 12 Mansfield Rd, Oxford OX1 3TA, UK.; 4 Centre for BioImaging Sciences, Department of Biological Sciences, National University of Singapore, 14 Science Drive 4, Singapore 117557, Singapore.; 5 Biomedical and X-ray Physics, Department of Applied Physics, AlbaNova University Center, KTH Royal Institute of Technology, SE-10691 Stockholm, Sweden.; 6 ARC Centre of Advanced Molecular Imaging, Department of Chemistry and Physics, La Trobe University, Melbourne, Victoria 3086, Australia.; 7 Division of Scientific Computing, Department of Information Technology, Science for Life Laboratory, Uppsala University, Lägerhyddsvägen 2 (Box 337), SE-751 05 Uppsala, Sweden.; 8 Department of Physics and Astronomy, Uppsala University, Box 516, SE-75120 Uppsala, Sweden.; 9 Institute of Physics, ELI Beamlines, Academy of Sciences of the Czech Republic, Na Slovance 2, CZ-18221 Prague, Czech Republic.; 10 Institute for Protein Research, Osaka University, Suita, Osaka 565-0871, Japan; 11 Department of Virology, Graduate School of Biomedical and Health Sciences, Hiroshima University, Hiroshima 734-8551, Japan; 12 Cryo-Electron Microscopy, Bijvoet Center for Biomolecular Research, Utrecht University, 3584 CH Utrecht, Netherlands; 13 Department of Physics, National University of Singapore, Singapore, Singapore.; 14 Linac Coherent Light Source, SLAC National Accelerator Laboratory, Menlo Park, CA 94025, USA.; 15 Chemical Sciences and Engineering Division, Argonne National Laboratory, Argonne, IL 60439, USA.; 16 Department of Physics and Astronomy, Northwestern University, Evanston, IL 60208, USA.; 17 Stanford PULSE Institute, SLAC National Accelerator Laboratory, Menlo Park, CA 94025, USA.; 18 Institut für Quantenoptik, Leibniz Universität Hannover, Welfengarten 1, 30167 Hannover, Germany.; 19 Institut für Optik und Atomare Physik, Technische Universität Berlin, 10623 Berlin, Germany.; 20 Condensed Matter Physics, Department of Physics, Chalmers University of Technology, Gothenburg, Sweden.; 21 NERSC, Lawrence Berkeley National Laboratory, Berkeley, CA 94720, USA.

## Abstract

The possibility of imaging single proteins constitutes an exciting challenge for x-ray lasers. Despite encouraging results on large particles, imaging small particles has proven to be difficult for two reasons: not quite high enough pulse intensity from currently available x-ray lasers and, as we demonstrate here, contamination of the aerosolized molecules by nonvolatile contaminants in the solution. The amount of contamination on the sample depends on the initial droplet size during aerosolization. Here, we show that, with our electrospray injector, we can decrease the size of aerosol droplets and demonstrate virtually contaminant-free sample delivery of organelles, small virions, and proteins. The results presented here, together with the increased performance of next-generation x-ray lasers, constitute an important stepping stone toward the ultimate goal of protein structure determination from imaging at room temperature and high temporal resolution.

## Teaser

Electrospray contaminant-free delivery of proteins to x-ray laser heralds the start of single-protein x-ray diffractive imaging.

## INTRODUCTION

Coherent diffractive imaging (*1*, *2*) with femtosecond ultrabright pulses from x-ray free-electron lasers (XFELs) has been proposed as a way to outrun key damage processes, allowing diffraction data to be recorded from a virtually undamaged particle before the particle is obliterated by the intense pulse (*3*, *4*). This opens new possibilities for the high-resolution imaging of biological samples without freezing, sectioning, or staining. Thus far, this method has not yet fulfilled its early promise of high-resolution subnanometer imaging. The scarcity of experimental time, demanding sample delivery requirements, and limited beam flux have been the main obstacles (*5*). These are slowly being tackled by the increase in performance and number of XFELs around the world, as well as improvements in sample delivery technology. Despite these challenges, the method has been successfully applied to large viruses, organelles, and even entire cells (*6*–*8*).

High-fluence XFEL beams, specialized detectors, low background noise, and efficient sample delivery into the XFEL focus were critical for the success of these pioneering studies (*6*, *7*, *9*). The most widely used injector for this approach, the Uppsala injector (*10*), generates a droplet aerosol by atomizing the sample solution with a gas-dynamic virtual nozzle (GDVN) (*11*). The volatile droplet components evaporate, leaving behind one aerosol particle for every occupied droplet. A skimmer removes excess aerosol carrier gas, and an aerodynamic lens focuses the aerosol to a narrow beam that is directed into the XFEL focus for imaging individual aerosol particles. While this injector has been used for imaging biological particles with diameters between 80 nm (*12*) and 2000 nm (*7*), imaging smaller particles has proven challenging (*13*, *14*). Particles appeared rounder and larger and showed a higher level of polydispersity than in solution (*7*, *13*, *14*).

It has been suspected that large and polydisperse initial droplets may be the cause for this size and shape mismatch (*14*). Nonvolatile contaminants are often unavoidable components of the sample solution, and the initial droplet size determines how much remains attached to the aerosolized particle after solvent evaporation. This problem is also known in electrospray (ES)–ionization mass spectrometry, as these contaminants degrade the mass spectral signal-to-noise ratio (*15*).

For droplet formation with GDVNs, a narrow cone-jet from the nozzle of a capillary is hydrodynamically tapered by a He sheath gas, up to the point at which the jet becomes unstable and breaks up into small droplets. This jet-atomization technique is efficient for the continuous creation of a large number of aerosol droplets with diameters of micrometers to submicrometers (*11*).

ES is an alternative jet-atomization technique (*16*, *17*). By applying a voltage to the liquid, the jet is squeezed into a Taylor cone, by electrostatic forces, without the requirement of exerting pressure by a sheath gas. ES has become a very powerful method to aerosolize biological particles with a wide range of sizes for examination by mass spectrometry (*18*, *19*) or differential mobility analysis (DMA) (*20*). Low flow rates and small droplets can be obtained, achieving gentle aerosolization with low contamination. A prerequisite for a stable Taylor cone is an inert and dielectric ambient gas that does not react and does not remove electrical charge from the liquid. A mixture of CO_2_ and N_2_ at a pressure of at least 800 mbar fulfills this requirement (*18*), which in our injector leads to a mass flow of 1.2 standard liters per minute (SLM) N_2_ and 0.15 SLM CO_2_. In contrast, the GDVN produces less than 0.5 SLM of He.

## RESULTS

### Characterization of droplet formation

**Fig. 1:**
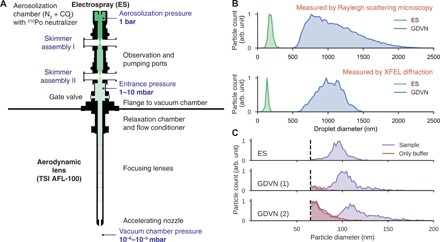
ES aerosol injector. (**A**) Design of the ES aerosol injector. In the aerosolization chamber, the ES nebulizer generates droplets that are neutralized with a ^210^Po alpha emitter. The ES nebulizer is operated in an atmosphere of N_2_ and CO_2_ at 1 bar. The aerosol is transported through two nozzle-skimmer assemblies, where excess gas is pumped away. At a reduced pressure of 1 to 10 mbar, the aerosol enters the aerosol lens stack, which focuses it to a narrow particle beam entering the experimental chamber, which is held at a pressure of 10^−6^ to 10^−5^ mbar to match requirements for XFEL imaging. (**B**) Size distributions of initial droplets for ES (green) and GDVN (blue) aerosols determined by RSM (top) and XFEL diffraction (bottom). The results of the two sizing methods are comparable within the limits of reproducibility expected for the manually manufactured nozzles and variations in operational parameters, such as pressures, voltage, and flow rate. (**C**) RSM size distributions of aerosolized particles from carboxysome sample (purple) and from its buffer solution (red). Data collected on electrosprayed particles are shown in the first panel (median, 95 nm; FWHM, 14 nm), and data collected on particles injected by GDVN at two different pressure configurations (Table 2) are shown in the second (median, 102 nm; FWHM, 17 nm) and third panels (median, 105 nm; FWHM, 17 nm). Dashed lines indicate the detection limit.

We modified the design of the Uppsala aerosol sample injector and substituted the GDVN with an ES aerosolizer. To reduce the increased mass flow from the dielectric gas, we added an additional nozzle-skimmer stage (Fig. 1A). The operational parameters for the GDVN aerosolizer are substantially different from the ES aerosolizer (Table 1). While our GDVNs are operated at liquid flow rates (*Q*) on the order of microliters per minute, our ES aerosolizer is operated at ~20 times lower flow rates. As the droplet volume (*V*) of the ES aerosolizer is ~300 times smaller than for GDVNs, ES produces droplets at ~15 times higher rate (*R* = *Q*/*V*). Theoretically, therefore, higher hit rates should be achievable by ES compared to GDVN aerosolization under usual conditions.

**Table 1:** Aerosolization parameters. Characteristic parameters for sample aerosolization with ES and a GDVN assuming an average droplet occupancy of 1.

	**Sample** **flow rate**	**Droplet** **size**	**Sample** **concentration**	**Particle rate**
ES	0.06 μl/min	150 nm	5 × 10^14^/ml	5.7 × 10^8^/s
GDVN	2 μl/min	1000 nm	2 × 10^12^/ml	0.6 × 10^8^/s

To compare droplet formation between ES and GDVN, we first determined the size distributions of initial droplets of the two aerosolizers (Fig. 1B) by measuring the size of particles that are formed when injecting sucrose solution (*21*). Sizes were measured by Rayleigh scattering microscopy (RSM) (*10*) and, in addition, by XFEL diffraction (*7*, *14*). The droplets generated with the GDVN span a wide range of diameters (500 to 2000 nm), whereas droplets generated with the ES aerosolizer are smaller and more monodisperse (100 to 200 nm).

For comparing bioparticle aerosols generated with the two injector designs, we selected carboxysomes as a biological test sample. Carboxysomes are polyhedral cell organelles that are heterogeneous in size with an average diameter of about 100 nm (*7*). Using RSM, we found that particles have, on average, larger diameters if aerosolized with a GDVN compared to ES (Fig. 1C, purple histograms). This observation confirms that the amount of nonvolatile contaminants that accumulate on the surface of aerosol particles increases with the size of the initial droplet. Furthermore, control measurements on only buffer (Fig. 1C, red histograms) revealed the presence of contaminant particles in the GDVN aerosols. These are likely aggregates of nonvolatile buffer remaining after solvent evaporation from empty droplets.

### Experimental tests at an XFEL

We tested the ES injector for x-ray imaging at the Atomic, Molecular, Optical Sciences (AMO) beamline at the Linac Coherent Light Source (LCLS). As test samples, we selected carboxysomes, tomato bushy stunt virus (TBSV) particles, and the protein ribulose-1,5-bisphosphate carboxylase/oxygenase (Rubisco, EC 4.1.1.39).

**Fig. 2:**
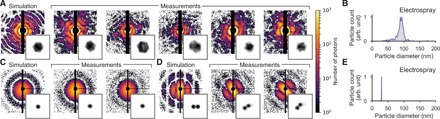
XFEL diffraction data of biological particles injected with the ES aerosol injector. (**A**) Simulated and measured diffraction patterns of carboxysomes and (**B**) their size distribution (median, 90 nm; FWHM, 13 nm) determined from the measured diffraction patterns. (**C and D**) Simulated and measured diffraction patterns of TBSV particles (C, singles; D, clusters of two) and (**E**) their size distribution (median, 30 nm; FWHM, 1 nm) determined from the measured diffraction patterns. Insets in (A), (C), and (D) show 2D projection images reconstructed from the respective diffraction patterns. The edge length of the insets corresponds to 220 nm.

In a previous study with GDVN aerosolization, we obtained high-quality diffraction images on carboxysomes, albeit most of the particles appeared round instead of icosahedral as would be expected (*7*). From the new diffraction data with ES aerosolization (5000 hits recorded within 7 min), we reconstructed projection images of carboxysomes (Fig. 2A) and determined the size distribution (Fig. 2B). Almost all particles matched projections of an icosahedral particle, and both the median and SD of the size distribution are in agreement with our RSM measurements (Fig. 1C). These results confirm that ES injection, in comparison to GDVN aerosolization, reduces the amount of nonvolatile contaminants.

TBSV particles are monodisperse with a diameter of about 35 nm. Despite their small size, 6000 high-quality diffraction patterns of single and double particles (Fig. 2, C and D) were collected within 1 hour of data collection. Particle clusters are expected because of the high sample concentration and the possibility of double occupancy of the droplets. The reconstructed projection images show the expected shape. The size distribution has a full-width at half-maximum (FWHM) smaller than 1 nm (Fig. 2E), which shows that ES did not alter the size distribution of the sample.

**Fig. 3:**
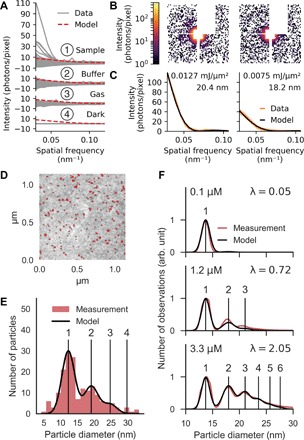
Injection of Rubisco proteins. (**A**) Radial averages of 14,361 background-subtracted diffraction patterns recorded during injection of sample (1), 14,343 during injection of buffer solution (2), 14,367 during injection of only gas (3), and 6993 during a dark run (4). (**B**) Diffraction patterns of two intense sample hits. (**C**) Radial averages (orange lines) of the diffraction patterns shown in (B) and fits (black lines) to a sphere model that best match the data. Light orange areas indicate the confidence intervals of the data (±1 SD). The fit values for intensity and sphere diameter are annotated. (**D**) STEM image of Rubisco proteins injected onto a TEM sample support film. Detected particles are highlighted in red. (**E**) The red histogram shows the distribution of particle diameters derived from (D). The black line shows the fit of our droplet occupancy model to the data. The good match indicates that the electrosprayed proteins were successfully transferred into the interaction region. (**F**) DMA data of electrosprayed Rubisco proteins at three concentrations. Our droplet occupancy model (black) was fitted to the measured size histograms (red). The agreement shows that, by changing concentration, we specifically control the protein cluster composition.

As a third test sample, we injected 11-nm-sized Rubisco proteins. The x-ray cross section for a Rubisco protein is about 30 times smaller than for a TBSV particle. In Fig. 3A, we compare the predicted signal (red dashed lines), using the measured incident peak intensity, to radially averaged diffraction data on injected Rubisco and respective control data on injected sample buffer solution, injection gas, and a dark run (solid lines in panels 1, 2, 3, and 4, respectively). The comparison shows that the predicted diffraction pattern for a single protein was too faint to exceed gas background fluctuations. Nevertheless, we found diffraction patterns that exceeded the amplitude of background fluctuations, and two examples are shown in Fig. 3B. From the diffraction images, we determined particle diameters matching the approximate size of a protein cluster of two to three particles (Fig. 3C).

Because of the weak scattering signal obtained with the pulse intensity that was available at the LCLS, we could not conclusively determine whether single Rubisco proteins were delivered into the interaction region. To answer this question, we injected Rubisco and deposited the injected particles for examination by scanning transmission electron microscopy (STEM) (Fig. 3D). We extracted the size distribution of deposited particles (Fig. 3E, red histogram) by integrating the areas of particles in the image. The size distribution matched a Poissonian droplet occupancy model (Fig. 3E, black line), which proves that we injected single Rubisco proteins into vacuum. We confirmed the validity of this model by measuring the size distribution of the same sample at a range of concentrations by DMA (Fig. 3F).

## DISCUSSION

We report successful single-particle imaging of 35-nm biological samples—substantially smaller than previously possible. Our adaptation of the Uppsala injector for ES was shown to decrease droplet sizes and was shown to enable delivery of single proteins into vacuum. Although this is not the first demonstration of ES injection for x-ray diffractive imaging (*9*), it represents a quantum leap toward single-protein imaging by achieving an increase of over two orders of magnitude in hit rate. This was achieved while decreasing the particle volume by three orders of magnitude, making them travel faster and therefore less likely to be hit (*10*). Furthermore, we injected isolated biological particles in contrast to the previous study, where the particles were surrounded by a thick layer of sucrose.

With this achievement, we overcome one of the major experimental hurdles that have hindered progress for XFEL imaging of small biological particles. For large particles, the smaller droplets of ES are also beneficial, as they reduce contamination from nonvolatile buffer components. As a result of the higher reproducibility of aerosolized particles, ES injection is also expected to increase attainable resolution in three-dimensional (3D) reconstructions.

Further development in lens stack design (*22*) and aerosolization geometry is expected to increase the particle transmission and decrease fluorescence and scattering background from injection gas that dominated the noise in our diffraction data. We anticipate that these diffraction data from single proteins will be possible to analyze using established 3D reconstruction methods (*12*, *23*). The results presented here, together with the increased x-ray flux and repetition rate of next-generation FEL facilities such as European XFEL and LCLS II, will constitute an important stepping stone toward the ultimate goal of protein structure determination from imaging at room temperature and high temporal resolution.

## MATERIALS AND METHODS

### Sample preparation

#### Sucrose solutions for aerosol droplet size determination

Initial droplet size distributions of ES and GDVN aerosols were determined by measuring the size distributions of particles generated by injecting sucrose solution. The particle diameter *d*_p_ is related to the initial droplet diameter *d*_0_ via the relation *d*_p_ = *d*_0_*c*^1/3^, where *c* is the volume concentration of sucrose. Sucrose concentrations were adjusted to achieve final particle sizes after solvent evaporation of around 100 nm, suitable for sizing by RSM and XFEL diffraction. For the RSM measurements, we used sucrose solutions at volume concentrations of 12% for ES and 0.1% for GDVN aerosolization, and for the XFEL diffraction measurements, we used 5% for ES and 0.1% for GDVN injection.

#### Carboxysome purification

Carboxysomes were purified from *Halothiobacillus neapolitanus* DMS15147 cells, as previously described in (*7*), with minor changes to the protocol with respect to the lysis of the cells (omitting the sonication step). After harvesting by centrifugation, the cells were resuspended in 50 ml of TEMB-lysozyme buffer [10 mM tris-HCl (pH 8.0), 10 mM MgCl_2_, 20 mM NaHCO_3_, 1 mM EDTA, and lysozyme (100 μg/ml; pH 8.0)]. The cell suspension was mixed with 50 ml of B-PER Bacterial Protein Extraction Reagent (Thermo Fisher Scientific) and incubated for about 10 min at room temperature on a rotary shaker. When the solution turned viscous, due to DNA release from broken cells, deoxyribonuclease I from bovine pancreas (Sigma-Aldrich) was added to a final concentration of 1 μg/ml. The suspension was incubated for an additional 30 min at room temperature on a rotary shaker. After pelleting the debris, the carboxysomes were purified by centrifugation and resuspension as described in (*7*). For ES injection, we used the purified sample at a concentration of about 10^13^ particles/ml in TEMB-lysozyme buffer (i.e., without exchanging the buffer). For GDVN injection, carboxysomes were buffer-exchanged by eluting the sample into 20 mM ammonium acetate solution (pH 7.5) using a PD MiniTrap G-25 column (GE Healthcare). This exchange was performed twice. We followed the same buffer exchange protocol for the control measurements in TEMB-lysozyme buffer.

#### TBSV purification

TBSV (strain BSV-3, American Type Culture Collection code PV-90) was propagated in *Nicotiana benthamiana* grown at 25°C under a 16-hour/8-hour light/dark cycle. Leaves were mechanically inoculated using carborundum and virus extract. At 6 to 8 days after infection, leaves that showed severe signs of infection were harvested and stored at −20°C. Frozen leaves, chilled with liquid nitrogen, were ground into a fine powder using a mortar and pestle and transferred into an ice-cooled BeadBeater (BioSpec Products Inc.; 2-mm zircona beads). Ice-cold extraction buffer [50 mM sodium phosphate (pH 5) and 1 mM tris(2-carboxyethyl)phosphine (TCEP)] was added to the ground leaf tissue in a volume to weight ratio of 5:1, before five rounds of 60-s/60-s on/off cycles. The solution was cleared from precipitated proteins and cell debris by centrifugation at 8000*g* for 30 min at 4°C. The supernatant was sequentially filtered using 5-, 0.8-, and 0.2-μm syringe filters. Virus particles were sedimented by ultracentrifugation at 100,000*g* for 2 hours at 4°C. The resulting pellets were carefully resuspended into native buffer [50 mM tris-HCl (pH 7.5) and 20 mM CaCl_2_] and cleared from any undissolved particulates by centrifugation for 1 min at 20,000*g*. The resuspended pellet was floated on a 15 to 60% preformed sucrose gradient (made using native buffer) and was subjected to rate-zonal centrifugation at 100,000*g* for 2 hours at 4°C. The virus particles could be seen as a band approximately one-third from the top of the tube when illuminated from the top. The band was recovered in fractions by pipetting and analyzed for ultraviolet absorption at 260 and 280 nm (NanoDrop; Thermo Fisher Scientific) and by SDS–polyacrylamide gel electrophoresis (PAGE). The sucrose was removed by dialysis into native buffer. Exchange into the injection buffer [25 mM ammonium acetate (pH 5)] was achieved by multiple rounds of sample dilution and subsequent concentration using a VivaSpin 10,000 MWCO (molecular weight cut-off) concentrator (Vivascience). The final particle concentration used for injection was 3 × 10^14^ to 5 × 10^14^ ml^−1^. Sample quality was verified by measuring size homogeneity and shape by dynamic light scattering (W130i; Avid Nano Ltd.) and negative-stain electron microscopy (FEI Quanta; Thermo Fisher Scientific).

#### Rubisco purification

*Spinacia oleracea* Rubisco was purified as previously described in (*24*). After long-term storage at −80°C, the sample was further purified by size-exclusion chromatography using a HiLoad 26/60 Superdex 200 (GE Healthcare) column attached to an NGC chromatography system (Bio-Rad). Separation was performed at 4°C, with a flow rate of 2 ml/min, in Superdex buffer [50 mM tris-Cl (pH 8.0), 100 mM NaCl, and 1 mM EDTA]. Peak fractions containing Rubisco identified by SDS-PAGE were pooled and concentrated using a VivaSpin 30,000 MWCO concentrator (Vivascience).

Purified *S. oleracea* Rubisco was incubated at room temperature for 30 min in the presence of equimolar 4-carboxy-d-arabinitol-1,5-bisphosphate (4-CABP), a reaction-intermediate analog that binds tightly and irreversibly to Rubisco active sites. 4-CABP binding induces a conformational change of a surface exposed loop to cover the active site of Rubisco, thereby reducing the structural heterogeneity of the sample (*24*).

Before injection, the protein was buffer-exchanged into ammonium acetate sample buffer [20 mM ammonium acetate (pH 7.97)] over a PD10 desalting column (GE Healthcare), as described above.

### Sample aerosolization

#### Gas-dynamic virtual nozzles

GDVNs were manufactured in-house according to the general design presented in (*11*). The generation of submicrometer droplets requires a large reduction in gas pressure around the liquid jet meniscus together with a low liquid flow rate. To achieve this, we used a “flush” geometry as described in (*25*) together with a 20–μm–inner diameter liquid capillary, whose tip was conically grinded at an attack angle of approximately 15° to 20°. Stable jets were achieved with liquid flow rates between 0.5 and 2 μl/min and an outer He sheath flow between 0.5 and 1.5 SLM.

#### Electrospray

The ES nebulizer was based on the design introduced in (*26*). The sample was supplied with 360–μm–outer diameter fused silica capillaries with inner diameter of 30 μm when injecting TBSV, sucrose, and Rubisco, while capillaries with an inner diameter of 40 μm were used when injecting carboxysomes. The capillaries were conically grinded at an attack angle of 30° until the tip of the capillary had a “plateau” with a diameter of 80 μm. During nebulization, the tip of the capillary was positioned approximately 1 mm away from a grounded orifice plate with an orifice diameter of 0.5 mm. The formation of a Taylor cone was achieved by applying a voltage of 2 to 3 kV to the sample inside the sample reservoir while the sample was flowing with a flow rate of 50 to 100 nl/min. The flow rate was achieved by applying an overpressure of 1 to 10 psi in the sample reservoir. To keep the Taylor cone stable, an influx of 0.15 liters/min CO_2_ + 1 liter/min N_2_ was necessary to avoid decharging of the liquid at the meniscus. The exact voltages and flow rates needed to achieve a stable Taylor cone vary with the conductivity of the sample. In this configuration, stable operation could be achieved with conductivities between 1700 and 7000 μS/cm. The charged droplets generated by the ES aerosolization were neutralized with a ^210^Po alpha source.

### Aerosol injection

Particles were delivered into the in-vacuum interaction region for GDVN aerosolization with the original and for ES aerosolization with the modified version of the Uppsala aerosol injector (*6*, *7*, *27*). Excess gas from the aerosolization process was removed in a nozzle-skimmer stage located between the aerosolization compartment and the aerodynamic lens stack. For GDVN aerosolization (*11*, *28*), a single nozzle-skimmer stage, with skimmer apertures of 0.3 and 0.6 mm, was required to reduce the gas load inside the aerodynamic lens stack. To accommodate the increased mass flow for ES aerosolization, we added a second nozzle-skimmer stage (Fig. 1A, skimmer assembly I), with 0.8-mm nozzle and 1-mm skimmer apertures. This additional stage was located upstream of the existing stage (Fig. 1A, skimmer assembly II). In both stages, the nozzle-skimmer distance was set such that the skimmer was located within the zone of silence (*27*) of the freely expanding gas exiting the nozzle.

### Particle sizing

#### Particle sizing by DMA

DMA measurements were carried out with the TSI3080 electrostatic classifier together with the TSI3081 differential mobility analyzer. The ES aerosol described above was used as input to the electrostatic classifier, while the size-selected particle output was detected with the TSI3786 condensed particle counter. In all, this system enabled detection and relative concentration measurements of particles 10 to 1000 nm in diameter.

#### Particle sizing by RSM

RSM data were acquired as described in (*10*). Size calibration was carried out with suspensions of Monodisperse Polystyrene Sphere Size Standards (Thermo Fisher Scientific; National Institute of Standards and Technology traceable size standard; refractive index, 1.5983). The calibration factors were rescaled on the basis of estimates for the refractive index of the respective particle species [carboxysomes, 1.4 (*29*); sucrose, 1.5376 (*30*)].

#### Particle size determination from XFEL diffraction intensities

The sizes of injected sucrose, carboxysome, and TBSV particles were determined by fitting the diffraction image of a uniform sphere model to the measured diffraction patterns (*14*). Table 2 lists the datasets that were used. Before fitting, the diffraction patterns were truncated below 0.5 photons, and pixels were binned (sucrose and TBSV data, 6 × 6; carboxysome data, 4 × 4). Throughout the fitting procedure, a binary mask was used that excluded hot, saturated, and shadowed pixels, and pixels at large diffraction angles where the signal from nonspherical objects is expected to deviate substantially from the sphere model. All run-specific parameters can be found in the files amol3116_sizing.csv and amol3416_sizing.csv under the open repository https://github.com/mhantke/electrospray_injection. In a last refinement step, we modified the fitting model to include an offset term to account for uniform background that was observed in the diffraction data. The sizing was carried out in an automated fashion together with a manual inspection of the fitted results and discarding of failed fits.

**Table 2:** Datasets used for this study. ID, inner diameter; n.a., not available.

**Measurement**	**Dataset** **name**	**Run** **#**	**Photon** **energy (eV)**	**Detector** **distance (mm)**	**Sample** **concentration**	**Liquid flow** **(μl/min)**	**Gas flow** **(SLM)**	**Capillary**	
								**ID** **(μm)**	**Voltage** **(kV)**
Sucrose (ES) (Fig. 1B, bottom panel)	AMO L3416	38	670	370	5 v/v %	0.06	CO_2_ 0.15 N_2_ 1.30	40	2.20
Sucrose (GDVN) (Fig. 1B, bottom panel)	AMO L3116	142	800	370	0.1 v/v %	0.7	He 0.4	n.a.	n.a.
Sucrose (ES) (Fig. 1B, top panel)	RSM	337	n.a.	n.a.	12 v/v %	0.06	CO_2_ 0.20 N_2_ 1.45	n.a.	n.a.
Sucrose (GDVN) (Fig. 1B, top panel)	RSM	385	n.a.	n.a.	0.1 v/v %	0.44	He 0.4	n.a.	n.a.
Carboxysomes (ES) (Fig. 1C, top panel)	RSM	301	n.a.	n.a.	1 × 10^13^ ml^−1^	0.06	CO_2_ 0.15 N_2_ 1.20	40	2.50
Carboxysomes (GDVN 1) (Fig. 1C, middle panel)	RSM	305	n.a.	n.a.	1 × 10^12^ ml^−1^	0.59	He 0.4	n.a.	n.a.
Carboxysomes (GDVN 2) (Fig. 1C, bottom panel)	RSM	309	n.a.	n.a.	1 × 10^12^ ml^−1^	0.59	He 0.6	n.a.	n.a.
Carboxysomes (ES) (Fig. 2, A to C )	AMO L3416	51–56	800	370	1 × 10^13^ ml^−1^	0.06	CO_2_ 0.15 N_2_ 1.30	40	2.15
TBSV (ES) (Fig. 2, C to E)	AMO L3416	132–135 137–142	800	259	3 × 10^14^ ml^−1^	0.06	CO_2_ 0.15 N_2_ 1.30	30	2.25
Rubisco (sample) (Fig. 3A, panel 1)	AMO L3416	252	800	130	8 × 10^14^ ml^−1^	0.06	CO_2_ 0.15 N_2_ 1.30	30	2.25
Rubisco (buffer) (Fig. 3A, panel 2)	AMO L3416	203	800	130	n.a.	0.06	CO_2_ 0.15 N_2_ 1.30	30	2.15
Rubisco (gas) (Fig. 3A, panel 3)	AMO L3416	256	800	130	n.a.	0.00	CO_2_ 0.15 N_2_ 1.30	n.a	n.a.
Rubisco (dark) (Fig. 3A, panel 4)	AMO L3416	257	800	130	n.a.	n.a.	n.a.	n.a.	n.a.

Rubisco particles were sized by fitting the radial diffraction intensities of a sphere model to the radially averaged diffraction intensities of the measurement. To validate our results, we checked that the incident intensity that resulted from the fit fell into the range of intensities expected for the x-ray beam focus (*7*). For this calculation, we assumed that the particles had a mass density of 1.35 g/cm^3^ and an atomic composition of H_86_C_52_N_13_O_15_S (*4*).

#### Particle sizing by electron microscopy

Rubisco particles exiting in a collimated beam from the aerosol injector were collected by streaking on a 400-mesh Cu F/C EM grid (Ted Pella Inc). The grid was then imaged without staining at ×240,000 magnification in a FEI Quanta FEG 650 using a STEM detector at an acquisition time of 1 μs and at an acceleration voltage of 30 kV.

### XFEL diffraction measurements

#### Data collection

XFEL diffraction data were collected inside the LAMP (laser applications in materials processing) chamber (*31*) at the AMO endstation (*32*) of the LCLS. The particle beam exiting from the Uppsala aerosol injector was intersected with the x-ray beam. The LCLS generated x-ray pulses of 1 to 2 mJ at a photon energy of 800 eV (wavelength, 1.55 nm) with a pulse duration of 170 fs and a peak fluence of 0.02 mJ/μm^2^ (*14*) at a repetition rate of 120 pulses/s. About 5% of the LCLS pulses were dumped (“BYKICK” mode) to continuously monitor the dark background. This means that the LCLS delivered effectively only about 114 pulses/s to the interaction region. Diffraction images were recorded synchronously with a pair of pnCCD area detector panels (*33*) operated in gain mode 5. The panels were placed at distances of 250 mm (TBSV data) and 370 mm (carboxysome and sucrose data). Each panel has a sensitive area of 76.8 mm × 38.4 mm with 1024 × 512 pixels. The direct beam and small-angle scattering passed through the gap between the panels. At a detector distance of 250 mm, the gap was 3.3 mm wide, and at a detector distance of 370 mm, it was 5.5 mm wide. Data were monitored live with the Hummingbird software package (*34*).

#### Data preprocessing

Diffraction data were preprocessed using the Hummingbird software package (*34*) and Psana (*35*). Configuration files (conf_preproc.py and conf_amol3416.py) can be downloaded from https://github.com/mhantke/electrospray_injection. The datasets that were used for analysis are listed in Table 2. Raw data were pedestal-subtracted using dark frames and rescaled to the unit of x-ray photons. Pedestal correction was followed by a three-step common mode subtraction procedure that was carried out for each panel individually, first for every quadrant (half panel), then for each fast, and finally for each slowly changing pixel dimension. Common mode is defined as the median pixel value of the selection of pixels that measure below 0.5 photons. For the faulty top-right quadrant, additionally ASIC (application-specific integrated circuit)–wise common mode subtractions were applied, first for the fast and then for the slowly changing pixel dimension. For certain runs (defined in amol3116_run_params.csv and amol3416_run_params.csv), all pixels of the inner one or two ASICs of the faulty quadrant were upscaled by a factor of 2. Detector geometry was applied by taking into account the relative position of the detector halves, the pnCCD readout timing issue for particular runs, and the column mismatch that was caused by a wiring error of the pnCCD chip. As hits, we selected those diffraction patterns that counted more than 3500 pixels measuring at least one photon and being located further than 200 pixels away from the center.

#### Data prediction

Diffraction data for carboxysomes, TBSV particles, and Rubisco proteins were simulated with the Condor software package (*36*). For Rubisco proteins, the electron density was estimated to be 0.43 Å^−3^ on the basis of a mass density of 1.35 g/cm^3^ and an atomic composition of H_86_C_52_N_13_O_15_S for proteins (*3*). The incident intensity was set to the measured peak fluence of 0.02 mJ/μm^2^.

#### Image reconstruction

For retrieving the phase of selected carboxysome and TBSV diffraction patterns and reconstructing 2D projection images, we used the Hawk software package (*37*). Before phasing, the diffraction patterns were truncated at 0.5 photons and binned to 128 × 128 images. We used a binary mask excluding hot, saturated, and shadowed pixels.

**Fig. 4:**
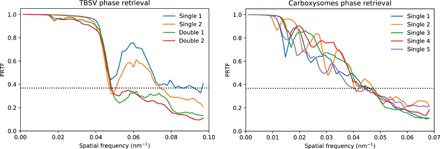
PRTFs for reconstructed projection images shown in Fig. 2 (A, C, and D). The dashed lines indicate the value *e*^−1^, often used as threshold for judging the reproducibly of the retrieved phases.

The support was initialized with a static spherical mask of radius slightly larger than the expected particle size. The iterative phase retrieval was performed with 1000 iterations of the relaxed averaged alternating reflections algorithm (*38*) (TBSV hits) or the hybrid input-output (HIO) algorithm (*39*) (carboxysome hits), followed by 1000 iterations of the error reduction algorithm (*39*) in both cases, enforcing the projected electron densities to be real and positive. The final reconstruction is an average of 100 independent reconstructions with a random initial guess for the phases. To check for reproducibility of the reconstructions, we calculated phase retrieval transfer functions (PRTFs) (Fig. 4).

#### Rubisco data analysis

Diffraction patterns were preprocessed as described above and then binned 16 × 16 pixels to improve the signal-to-noise ratio. Then, pixel values below the background floor of half a photon were set to zero to reduce the background from gas fluorescence and visible light, and all other values were rounded to the closest integer value. For every pixel, the variance and the mean value were calculated from the buffer run. Pixels for which the ratio of variance and mean value deviated by less than 0.3 from 1 were identified as good pixels because of the indication that their values followed Poisson statistics. Pixels that did not fall into this category were masked out. The mask was extended manually to exclude the halo of the direct beam and the edges of the detector quadrants. Last, images were background-corrected by subtraction of the median readout value for every pixel, respectively.

### Droplet occupancy model

The droplet occupancy by particles during droplet formation was modeled as a Poissonian process. The expectation value λ for the occupancy *n* of a droplet is given by the product of particle concentration in solution and droplet volume. For multiply occupied droplets (*n* > 1), the particles stick together and form a (nonspecific) complex. We assumed that the diameter of the complex *d_n_* does not grow as *d_n_*~*n*^1/3^ because the new complex will be most likely less compact than a sphere. Instead, we fitted the distributions shown in Fig. 3, E and F, by using the scaling law *d_n_*~*n*^1/*a*^, with the free parameter *a < 3*. We obtained *a* = 1.57 for the deposited proteins imaged by STEM and *a* = 2.56 for the DMA data.

## References

[1] Miao J., Charalambous P., Kirz J., Sayre D., Extending the methodology of x-ray crystallography to allow imaging of micrometre-sized non-crystalline specimens. Nature 400, 342–344 (1999).

[2] Miao J., Ishikawa T., Robinson I. K., Murnane M. M., Beyond crystallography: Diffractive imaging using coherent x-ray light sources. Science 348, 530–535 (2015). 25931551 10.1126/science.aaa1394

[3] Neutze R., Wouts R., van der Spoel D., Weckert E., Hajdu J., Potential for biomolecular imaging with femtosecond x-ray pulses. Nature 406, 752–757 (2000). 10963603 10.1038/35021099

[4] Bergh M., Huldt G., Tîmneanu N., Maia F. R. N. C., Hajdu J., Feasibility of imaging living cells at subnanometer resolutions by ultrafast x-ray diffraction. Q. Rev. Biophys. 41, 181–204 (2008). 19079804 10.1017/S003358350800471X

[5] Aquila A., Barty A., Bostedt C., Boutet S., Carini G., dePonte D., Drell P., Doniach S., Downing K. H., Earnest T., Elmlund H., Elser V., Gühr M., Hajdu J., Hastings J., Hau-Riege S. P., Huang Z., Lattman E. E., Maia F. R. N. C., Marchesini S., Ourmazd A., Pellegrini C., Santra R., Schlichting I., Schroer C., Spence J. C. H., Vartanyants I. A., Wakatsuki S., Weis W. I., Williams G. J., The linac coherent light source single particle imaging road map. Struct. Dyn. 2, 041701 (2015). 26798801 10.1063/1.4918726 PMC4711616

[6] Seibert M. M., Ekeberg T., Maia F. R. N. C., Svenda M., Andreasson J., Jönsson O., Odić D., Iwan B., Rocker A., Westphal D., Hantke M., DePonte D. P., Barty A., Schulz J., Gumprecht L., Coppola N., Aquila A., Liang M., White T. A., Martin A., Caleman C., Stern S., Abergel C., Seltzer V., Claverie J.-M., Bostedt C., Bozek J. D., Boutet S., Miahnahri A. A., Messerschmidt M., Krzywinski J., Williams G., Hodgson K. O., Bogan M. J., Hampton C. Y., Sierra R. G., Starodub D., Andersson I., Bajt S., Barthelmess M., Spence J. C. H., Fromme P., Weierstall U., Kirian R., Hunter M., Doak R. B., Marchesini S., Hau-Riege S. P., Frank M., Shoeman R. L., Lomb L., Epp S. W., Hartmann R., Rolles D., Rudenko A., Schmidt C., Foucar L., Kimmel N., Holl P., Rudek B., Erk B., Hömke A., Reich C., Pietschner D., Weidenspointner G., Strüder L., Hauser G., Gorke H., Ullrich J., Schlichting I., Herrmann S., Schaller G., Schopper F., Soltau H., Kühnel K.-U., Andritschke R., Schröter C.-D., Krasniqi F., Bott M., Schorb S., Rupp D., Adolph M., Gorkhover T., Hirsemann H., Potdevin G., Graafsma H., Nilsson B., Chapman H. N., Hajdu J., Single mimivirus particles intercepted and imaged with an x-ray laser. Nature 470, 78–81 (2011). 21293374 10.1038/nature09748 PMC4038304

[7] Hantke M. F., Hasse D., Maia F. R. N. C., Ekeberg T., John K., Svenda M., Loh N. D., Martin A. V., Timneanu N., Larsson D. S. D., van der Schot G., Carlsson G. H., Ingelman M., Andreasson J., Westphal D., Liang M., Stellato F., DePonte D. P., Hartmann R., Kimmel N., Kirian R. A., Seibert M. M., Mühlig K., Schorb S., Ferguson K., Bostedt C., Carron S., Bozek J. D., Rolles D., Rudenko A., Epp S., Chapman H. N., Barty A., Hajdu J., Andersson I., High-throughput imaging of heterogeneous cell organelles with an x-ray laser. Nat. Photon. 8, 943–949 (2014).

[8] van der Schot G., Svenda M., Maia F. R. N. C., Hantke M., DePonte D. P., Seibert M. M., Aquila A., Schulz J., Kirian R., Liang M., Stellato F., Iwan B., Andreasson J., Timneanu N., Westphal D., Almeida F. N., Odic D., Hasse D., Carlsson G. H., Larsson D. S. D., Barty A., Martin A. V., Schorb S., Bostedt C., Bozek J. D., Rolles D., Rudenko A., Epp S., Foucar L., Rudek B., Hartmann R., Kimmel N., Holl P., Englert L., Loh N.-T. D., Chapman H. N., Andersson I., Hajdu J., Ekeberg T., Imaging single cells in a beam of live cyanobacteria with an x-ray laser. Nat. Commun. 6, 5704 (2015). 25669616 10.1038/ncomms6704

[9] Bogan M. J., Benner W. H., Boutet S., Rohner U., Frank M., Barty A., Seibert M. M., Maia F., Marchesini S., Bajt S., Woods B., Riot V., Hau-Riege S. P., Svenda M., Marklund E., Spiller E., Hajdu J., Chapman H. N., Single particle x-ray diffractive imaging. Nano Lett. 8, 310–316 (2008). 18095739 10.1021/nl072728k

[10] Hantke M. F., Bielecki J., Kulyk O., Westphal D., Larsson D. S. D., Svenda M., Reddy H. K. N., Kirian R. A., Andreasson J., Hajdu J., Maia F. R. N. C., Rayleigh-scattering microscopy for tracking and sizing nanoparticles in focused aerosol beams. IUCrJ 5, 673–680 (2018). 10.1107/S2052252518010837 PMC6211534 30443352

[11] DePonte D. P., Weierstall U., Schmidt K., Warner J., Starodub D., Spence J. C. H., Doak R. B., Gas dynamic virtual nozzle for generation of microscopic droplet streams. J. Phys. D Appl. Phys. 41, 195505 (2008).

[12] Kurta R. P., Donatelli J. J., Yoon C. H., Berntsen P., Bielecki J., Daurer B. J., DeMirci H., Fromme P., Hantke M. F., Maia F. R. N. C., Munke A., Nettelblad C., Pande K., Reddy H. K. N., Sellberg J. A., Sierra R. G., Svenda M., van der Schot G., Vartanyants I. A., Williams G. J., Xavier P. L., Aquila A., Zwart P. H., Mancuso A. P., Correlations in scattered x-ray laser pulses reveal nanoscale structural features of viruses. Phys. Rev. Lett. 119, 158102 (2017). 29077445 10.1103/PhysRevLett.119.158102 PMC5757528

[13] Kassemeyer S., Steinbrener J., Lomb L., Hartmann E., Aquila A., Barty A., Martin A. V., Hampton C. Y., Bajt S., Barthelmess M., Barends T. R. M., Bostedt C., Bott M., Bozek J. D., Coppola N., Cryle M., DePonte D. P., Doak R. B., Epp S. W., Erk B., Fleckenstein H., Foucar L., Graafsma H., Gumprecht L., Hartmann A., Hartmann R., Hauser G., Hirsemann H., Hömke A., Holl P., Jönsson O., Kimmel N., Krasniqi F., Liang M., Maia F. R. N. C., Marchesini S., Nass K., Reich C., Rolles D., Rudek B., Rudenko A., Schmidt C., Schulz J., Shoeman R. L., Sierra R. G., Soltau H., Spence J. C. H., Starodub D., Stellato F., Stern S., Stier G., Svenda M., Weidenspointner G., Weierstall U., White T. A., Wunderer C., Frank M., Chapman H. N., Ullrich J., Strüder L., Bogan M. J., Schlichting I., Femtosecond free-electron laser x-ray diffraction data sets for algorithm development. Opt. Express 20, 4149–4158 (2012). 22418172 10.1364/OE.20.004149

[14] Daurer B. J., Okamoto K., Bielecki J., Maia F. R. N. C., Mühlig K., Seibert M. M., Hantke M. F., Nettelblad C., Benner W. H., Svenda M., Tîmneanu N., Ekeberg T., Loh N. D., Pietrini A., Zani A., Rath A. D., Westphal D., Kirian R. A., Awel S., Wiedorn M. O., van der Schot G., Carlsson G. H., Hasse D., Sellberg J. A., Barty A., Andreasson J., Boutet S., Williams G., Koglin J., Andersson I., Hajdu J., Larsson D. S. D., Experimental strategies for imaging bioparticles with femtosecond hard x-ray pulses. IUCrJ 4, 251–262 (2017). 10.1107/S2052252517003591 PMC5414399 28512572

[15] Hernández H., Robinson C. V., Determining the stoichiometry and interactions of macromolecular assemblies from mass spectrometry. Nat. Protoc. 2, 715–726 (2007). 17406634 10.1038/nprot.2007.73

[16] Yamashita M., Fenn J. B., Electrospray ion source. Another variation on the free-jet theme. J. Phys. Chem. 88, 4451–4459 (1984).

[17] Gañán-Calvo A. M., Montanero J. M., Revision of capillary cone-jet physics: Electrospray and flow focusing. Phys. Rev. E Stat. Nonlin. Soft Matter Phys. 79, 066305 (2009). 19658592 10.1103/PhysRevE.79.066305

[18] Fenn J. B., Mann M., Meng C. K., Wong S. F., Whitehouse C. M., Electrospray ionization for mass spectrometry of large biomolecules. Science 246, 64–71 (1989). 2675315 10.1126/science.2675315

[19] Tito M. A., Tars K., Valegard K., Hajdu J., Robinson C. V., Electrospray time-of-flight mass spectrometry of the intact MS2 virus capsid. J. Am. Chem. Soc. 122, 3550–3551 (2000).

[20] Kaufman S. L., Skogen J. W., Dorman F. D., Zarrin F., Lewis K. C., Macromolecule analysis based on electrophoretic mobility in air: Globular proteins. Anal. Chem. 68, 1895–1904 (1996). 21619100 10.1021/ac951128f

[21] Zarrin F., Kaufman S. L., Socha J. R., Droplet size measurements of various nebulizers usingdifferential electrical mobility particle sizer. J. Aerosol Sci. 22, S343–S346 (1991).

[22] Roth N., Awel S., Horke D. A., Küpper J., Optimizing aerodynamic lenses for single-particle imaging. J. Aerosol Sci. 124, 17–29 (2018).

[23] Ekeberg T., Svenda M., Abergel C., Maia F. R. N. C., Seltzer V., Claverie J.-M., Hantke M., Jönsson O., Nettelblad C., van der Schot G., Liang M., DePonte D. P., Barty A., Seibert M. M., Iwan B., Andersson I., Loh N. D., Martin A. V., Chapman H., Bostedt C., Bozek J. D., Ferguson K. R., Krzywinski J., Epp S. W., Rolles D., Rudenko A., Hartmann R., Kimmel N., Hajdu J., Three-dimensional reconstruction of the giant mimivirus particle with an x-ray free-electron laser. Phys. Rev. Lett. 114, 098102 (2015). 25793853 10.1103/PhysRevLett.114.098102

[24] Andersson I., Tjäder A.-C., Cedergren-Zeppezauer E., Brändén C.-I., Crystallization and preliminary x-ray studies of spinach ribulose 1,5-bisphosphate carboxylase/oxygenase complexed with activator and a transition state analogue. J. Biol. Chem. 258, 14088–14090 (1983). 6580292

[25] Gañán-Calvo A. M., DePonte D. P., Herrada M. A., Spence J. C. H., Weierstall U., Doak R. B., Liquid capillary micro/nanojets in free-jet expansion. Small 6, 822–824 (2010). 20213649 10.1002/smll.200901786

[26] Chen D.-R., Pui D. Y. H., Kaufman S. L., Electrospraying of conducting liquids for monodisperse aerosol generation in the 4 nm to 1.8 μm diameter range. J. Aerosol Sci. 26, 963–977 (1995).

[27] Campargue R., Progress in overexpanded supersonic jets and skimmed molecular beams in free-jet zones of silence. J. Phys. Chem. 88, 4466–4474 (1984).

[28] Gañán-Calvo A. M., Generation of steady liquid microthreads and micron-sized monodisperse sprays in gas streams. Phys. Rev. Lett. 80, 285–288 (1998).

[29] Choi W., Fang-Yen C., Badizadegan K., Oh S., Lue N., Dasari R. R., Feld M. S., Tomographic phase microscopy. Nat. Methods 4, 717–719 (2007). 17694065 10.1038/nmeth1078

[30] *Handbook of Chemistry and Physics*, R. C. Weast, Ed. (CRC Press Inc., ed. 60, 1979), p. C-503.

[31] Osipov T., Bostedt C., Castagna J.-C., Ferguson K. R., Bucher M., Montero S. C., Swiggers M. L., Obaid R., Rolles D., Rudenko A., Bozek J. D., Berrah N., The LAMP instrument at the Linac Coherent Light Source free-electron laser. Rev. Sci. Instrum. 89, 035112 (2018). 29604777 10.1063/1.5017727

[32] Ferguson K. R., Bucher M., Bozek J. D., Carron S., Castagna J.-C., Coffee R., Curiel G. I., Holmes M., Krzywinski J., Messerschmidt M., Minitti M., Mitra A., Moeller S., Noonan P., Osipov T., Schorb S., Swiggers M., Wallace A., Yin J., Bostedt C., The atomic, molecular and optical science instrument at the Linac Coherent Light Source. J. Synchrotron Radiat. 22, 492–497 (2015). 25931058 10.1107/S1600577515004646 PMC4416665

[33] Strüder L., Epp S., Rolles D., Hartmann R., Holl P., Lutz G., Soltau H., Eckart R., Reich C., Heinzinger K., Thamm C., Rudenko A., Krasniqi F., Kühnel K.-U., Bauer C., Schröter C.-D., Moshammer R., Techert S., Miessner D., Porro M., Hälker O., Meidinger N., Kimmel N., Andritschke R., Schopper F., Weidenspointner G., Ziegler A., Pietschner D., Herrmann S., Pietsch U., Walenta A., Leitenberger W., Bostedt C., Möller T., Rupp D., Adolph M., Graafsma H., Hirsemann H., Gärtner K., Richter R., Foucar L., Shoeman R. L., Schlichting I., Ullrich J., Large-format, high-speed, x-ray pnCCDs combined with electron and ion imaging spectrometers in a multipurpose chamber for experiments at 4th generation light sources. Nucl. Instrum. Methods Phys. Res. A 614, 483–496 (2010).

[34] Daurer B. J., Hantke M. F., Nettelblad C., Maia F. R. N. C., Hummingbird: Monitoring and analyzing flash x-ray imaging experiments in real time. J. Appl. Cryst. 49, 1042–1047 (2016). 27275147 10.1107/S1600576716005926 PMC4886990

[35] Damiani D., Dubrovin M., Gaponenko I., Kroeger W., Lane T. J., Mitra A., O'Grady C. P., Salnikov A., Sanchez-Gonzalez A., Schneider D., Yoon C. H., Linac Coherent Light Source data analysis using psana. J. Appl. Cryst. 49, 672–679 (2016).

[36] Hantke M. F., Ekeberg T., Maia F. R. N. C., *Condor*: A simulation tool for flash x-ray imaging. J. Appl. Cryst. 49, 1356–1362 (2016). 27504081 10.1107/S1600576716009213 PMC4970500

[37] Maia F. R. N. C., Ekeberg T., van der Spoel D., Hajdu J., Hawk: The image reconstruction package for coherent x-ray diffractive imaging. J. Appl. Cryst. 43, 1535–1539 (2010).

[38] Luke D. R., Relaxed averaged alternating reflections for diffraction imaging. Inverse Probl. 21, 37–50 (2005).

[39] Fienup J. R., Phase retrieval algorithms: A comparison. Appl. Optics 21, 2758–2769 (1982). 10.1364/AO.21.002758 20396114

